# Bringing a Gene-Activated Bone Substitute Into Clinical Practice: From Bench to Bedside

**DOI:** 10.3389/fbioe.2021.599300

**Published:** 2021-02-04

**Authors:** Ilia Y. Bozo, Alexey Y. Drobyshev, Nikolay A. Redko, Vladimir S. Komlev, Artur A. Isaev, Roman V. Deev

**Affiliations:** ^1^Department of Maxillofacial Surgery, A. I. Burnazyan Federal Medical Biophysical Center, Federal Medical Biological Agency of Russia, Moscow, Russia; ^2^Histograft, LLC, Moscow, Russia; ^3^Department of Maxillofacial and Plastic Surgery, A. I. Yevdokimov Moscow State University of Medicine and Dentistry, Moscow, Russia; ^4^A. A. Baikov Institute of Metallurgy and Materials Science, Russian Academy of Sciences, Moscow, Russia; ^5^Human Stem Cells Institute, Moscow, Russia; ^6^Department of Pathology, I. I. Mechnikov North-Western State Medical University, Saint Petersburg, Russia

**Keywords:** gene-activated matrix, bone substitute, octacalcium phosphate, osteogenesis, plasmid DNA, vascular endothelial growth factor, clinical trial

## Abstract

Bone grafting and reconstruction are still challenging in clinical practice because of the limitations of bone autografts and the drawbacks of currently approved bone substitutes. We thus developed a gene-activated bone substitute based on octacalcium phosphate and naked plasmid DNA carrying the vascular endothelial growth factor gene. This advanced combined therapy medicinal product had no cytotoxic effects *in vitro*, slightly decreased bone marrow mesenchymal stromal cell (MSC) doubling time, and was characterized by a prolonged level of gene construct delivery *in vivo* in a luciferase bioimaging assay. In the model of critically sized cranial bone defects in rabbits, the gene-activated matrix increased bone tissue formation through angiogenesis induction. After preclinical studies, we conducted an open-label non-randomized clinical trial (NCT03076138). The primary study outcome was the proportion of patients with newly formed bone tissue within the surgical area as measured by computed tomography within 6 months after surgery. The main secondary outcomes included frequencies of adverse events (AEs) and serious adverse events (SAEs) as well as the surgical failure rate. After completing the clinical trial, the patients had dental implants placed in the bone grafting area, and trephine biopsy samples were collected. In total, 20 patients with alveolar ridge atrophy (*n* = 16) and jaw bone defects (*n* = 4) were enrolled in the study. There were no AEs or SAEs during the clinical trial or the follow-up period (30 months). In all patients, newly formed tissues with a bone density of 908.13 ± 114.40 HU were detected within the zone of bone grafting. There were no significant differences between the subgroups of patients with atrophy and bone defects: 915.28 ± 125.85 and 879.56 ± 48.36 HU, respectively (*p* = 0.60). Histological analysis showed that the bone grafting area comprised newly formed bone tissue with some fragments of the gene-activated bone substitute partially resorbed and integrated with bone, without fibrous tissue in between. The preclinical data and clinical trial results proved the feasibility, safety, and efficacy of the investigated material for jaw bone grafting, allowing us to bring the world's first gene-activated bone substitute from bench to bedside.

## Introduction

Bone tissue has significant potential for reparative regeneration. However, with “osteogenic insufficiency,” this process can take a long time and often does results in incomplete bone healing, despite the use of current surgical technologies. Osteogenic insufficiency is a pathological condition associated with low activity of systemic or local osteoinductive factors and/or a lack of cambial cells in the bone lesion area; therefore, the natural course of reparative osteogenesis may not provide complete histotypic and organotypic recovery (Deev et al., 2015). Therefore, treatment of patients with large bone defects, delayed consolidation, and non-unions remains extremely challenging and is followed by a prolonged loss of working ability, decreased quality of life, and even disability. Successful treatment requires restoration of the lost cambial reserve and/or osteoinductive factors, and classically involves the use of bone autografts, as the “golden standard.” However, well-known limitations and disadvantages of this approach (Baldwin et al., 2019) predetermine the development of acceptable alternatives, among which activated bone substitutes are quite promising (Deev et al., 2015).

Alveolar ridge atrophy resulting from edentulism and lack of a necessary bone load is specific for oral and maxillofacial surgery. In this case, rational prosthetics, especially based on dental implants, require a local bone volume increase that can only be achieved using bone grafting procedures. According to the Straumann Group (Germany), more than 12 million dental implants are placed worldwide, with bone grafting required in one-fourth of the cases (Straumann Holding AG, 2018). Certain atrophies, for example, in height or in height and width simultaneously, are so complicated that modern guidelines do not recommend the use of bone grafts and the available substitutes for clinical use because of their low efficiency compared to autografts (Cordaro and Terheyden, 2015).

However, activated bone substitutes that deliver living cells, growth factors, or gene constructs encoding them can change this concept. For example, bone morphogenetic proteins have already been applied as biologically active substances for combination products in spinal fusion and bone grafting in oral surgery (Sheikh et al., 2015). Another promising candidate is vascular endothelial growth factor (VEGF), especially the VEGF-A165 isoform. VEGF is a well-known angiogenic agent that induces activation, migration, proliferation, and differentiation of endotheliocytes and their progenitor cells (Bhattacharya et al., 2009). Additionally, VEGF *via* a specific type 2 receptor, directly stimulates the proliferation and migration of osteogenic cells (Yang et al., 2012); some intracrine mechanisms have also been found for VEGF to be involved in the regulation of osteogenic differentiation (Liu et al., 2012).

Another important part of activated bone substitutes is a biocompatible scaffold that contributes to bone regeneration with both osteoconductive effects and biologically active component delivery. Different calcium phosphates are widely used for this purpose (Eliaz and Metoki, 2017). Among them, octacalcium phosphate (OCP) has been shown as a possible precursor for bone mineral components, capable of facilitating osteogenic cell differentiation and an effective scaffold for cell delivery (Komlev et al., 2014). Its biocompatibility and optimal osteoconductive capacities have been recently shown by several scientific groups (Shelton et al., 2006; Suzuki et al., 2006; Barinov and Komlev, 2010; Komlev et al., 2014).

From osteoinductive materials, gene-activated matrices belonging to combination products in the FDA terms (21 CFR part 3) and consisting of bioresorbable scaffolds and gene constructs encoding any therapeutic protein, have been the least studied and have no precedent of clinical translation prior to this study.

Considering the crucial role of VEGF-A in reparative osteogenesis, the osteoconductive potential of OCP, and our experience in gene-therapeutic drug development and clinical translation (Deev et al., 2018), we aimed to investigate and clinically translate a gene-activated bone substitute based on an OCP scaffold and *VEGFA165*-carrying plasmid DNA (pDNA-*VEGF*).

## Materials and Methods

### Gene-Activated Bone Substitute

The gene-activated bone substitute (OCP/pDNA-*VEGF)* comprises OCP granules (with diameters of 150–500 μm) with molecules of pDNA-*VEGF* (under the CMV promoter, the plasmid map is presented in Supplementary Figure 1), the active substance in Neovasculgen (HSCI, Russia), at 100 μg/g of OCP. OCP was synthesized according to a previously published protocol (Komlev et al., 2014). pDNA-*VEGF* was purchased from the Neovasculgen manufacturer for research and development purposes. OCP and pDNA-*VEGF* were combined using the original technology described in patent application US 20190224379A1. Briefly, OCP samples were incubated with 0.5 M sodium phosphate monobasic dihydrate (NaH_2_PO_4_.2H_2_O, Chimmed, Russia) at 37°C with constant shaking for 10 h, washed in a 10 mM solution of NaH_2_PO_4_.2H_2_O and sterilized by autoclaving. Next, pDNA-*VEGF* solution at a concentration of 100 μg/mL was applied in a volume of 1.0 mL−1.0 g of OCP samples, and the materials were incubated at 37°C with constant shaking for 10 h. A test batch of sterile lyophilized gene-activated bone substitute at 1.0 and 0.5 g was produced, stored in glass vials, and kept at a temperature of 2–6°C. The final dosage of pDNA-*VEGF* was 100 μg/g (≅1.0 cm^3^) of OCP.

### *In vitro* Biocompatibility Assay

Bone marrow mesenchymal stromal cell (MSC) culture was provided by the Burnazyan Federal Medical Biophysical Center. MSCs were seeded at a density of 5 × 10^4^ cells/well in a 12-well plate in DMEM (Invitrogen, USA) supplemented with 10% FBS, and were cultured for 24 h. Then, 50 mg of the investigated materials (OCP or OCP/pDNA-*VEGF*) were added per well using Transwell inserts with 3.0 μm pores (Corning, USA). After 72 h, co-incubation was stopped and 0.5 μg/mL of fluorescein diacetate (FDA, Ex./Em. = 490/520 nm, Invitrogen, USA) and 1 μg/mL propidium iodide (PI, Ex./Em. = 535/617 nm, Invitrogen, USA) were added to each well and incubated for 5 min in the dark to assess cell viability. The cell cultures were then washed thrice with PBS (Life Technologies Corp., USA). OCP-free cell cultures with and without pDNA-*VEGF* solution were used as controls. All experiments were performed in triplicate. Cell cultures were visually evaluated using an Olympus CKX53 microscope (Olympus Corp., Japan). Fluorescence was measured using a SpectraMax M5 Multi-Mode Microplate Reader (Molecular Devices, USA).

Additionally, the total number of cells was used for calculating the MSCs doubling time using the following formula:

$$\begin{array}{l} \text{Td}={\text{T}}_{\text{x}}\;\times \text{ ln}\left(2\right)/\text{ln }\left({\text{N}}_{\text{x}}/{\text{N}}_{0}\right), \end{array}$$

where Td – cell population doubling time, T_x_ – cultivation time, N_x_ – number of cells at T_x_ time point, and N_0_ – the initial number of cells.

### *In vivo* Plasmid DNA Delivery Assessment

All *in vivo* studies were performed in compliance with the national laws and policies for animal care. Balb/c mice weighing ~30 g (*n* = 18) were used in the experiment. The animals were divided into six groups (three mice per group) and received one of the following materials: gene-activated OCP with a reporter plasmid DNA encoding firefly luciferase gene (pDNA-*Luc*) instead of pDNA-*VEGF* (OCP/pDNA-*Luc*, 0.25 cm^3^, test group); OCP samples (0.25 cm^3^, negative control 1); OCP/pDNA-*VEGF* (0.25 cm^3^, negative control 2); a solution of pDNA-*Luc* (positive control); gene-activated hydrogel based on alginate and pDNA-*Luc* (0.25 cm^3^, test group 2); and gene-activated hydrogel based on hyaluronic acid delivering pDNA-*Luc* (0.25 cm^3^, test group 3). The latter two groups were used to compare the dynamics of *in vivo* cell transfection between the solid scaffold-based and hydrogel-based technologies. pDNA-*Luc* was combined with either solid or hydrogel scaffolds at a concentration of 100 μg/g (1.0 mL) of the scaffold.

Under infiltration anesthesia with 1% Lidocaine solution at 2 mL and intramuscular sedation with Zoletil 100 solution at 10 mg/kg, a 5 mm median skin incision was made in the back of each mouse, and a subcutaneous pocket was formed on the right side for implanting the solid materials (in a volume of 0.25 cm^3^), whereas hydrogels and the solution of pDNA-*Luc* were injected without subcutaneous dissection (in a volume of 0.25 cm^3^). In all groups, the dose of plasmid DNA was 25 μg. On days 1, 7, 14, and 28, a solution of D-luciferin sodium salt (Lumtech, Russia) at 150 mg/kg body weight was intraperitoneally injected into animals under Zoletil 100 solution (10 mg/kg) sedation. After 10 min, the animals were placed in a an IVIS Spectrum chamber (PerkinElmer, Inc., USA), and the luminescence signal (p/s/cm^2^/sr) was recorded for 1 min. Before D-luciferin sodium salt administration, mice were kept in the dark for 1 h to minimize background luminescence.

### Evaluation of Gene-Activated Bone Substitute in A Critically Sized Bone Defect Model

Male Chinchilla rabbits weighing 2.0–2.5 kg (*n* = 18) were used in the study and were divided into two cohorts: animals with bone grafting (*n* = 12) and empty defects (*n* = 6). Two identical circular full-thickness defects (10 mm in diameter) in both parietal bones were made in each animal, using trephine with an external diameter of 10 mm under 1% Lidocaine solution at 3 ml and sedation with Zoletil 100 solution at 10 mg/kg. The *dura mater* was preserved in all cases. OCP/pDNA-*VEGF* was implanted into the defect on the right side (test group, 12 defects), OCP without plasmid DNA was implanted on the left side (control group 1, 12 defects) in the bone grafting cohort. The amount of implanted material was 0.025 cm^3^ per defect. Fixation of granular materials within a bone defect was achieved by periosteum suturing. In the cohort with empty defects, no materials were implanted, that is, only blood clot filled the defects (control group 2, 12 defects). Animals were sacrificed at 30, 60, and 90 days after surgery (3 and 2 rabbits from cohorts 1 and 2, respectively, at each time point), their resected cranial bones were fixed in 10% neutral formalin solution for 7 days. The materials were then evaluated by cone-beam computed tomography (CT) using 3D Accuitomo 170 (J. Morita Corporation, Japan) in the same scanning mode and parameters (voxel size 0.08 mm, 80 kV, and 2 mA). The CT scans were analyzed using the standard tools in Planmeca Romexis viewer software (Planmeca Oy, Finland). Newly formed tissue density (in Hounsfield units, HU) was measured in the axial plane within the previously made bone defect using the “region of interest” tool with circular shape and diameter 10 mm (78.54 mm^2^). For histological analysis, cranial bones were decalcified in a Biodec R solution (Bio-optica, Italy), and 5 μm thick sections passing through the central part of the defect in the frontal plane were prepared; the sections were stained using hematoxylin and eosin, and a Masson-Goldner staining kit (Sigma-Aldrich, USA). To evaluate the angiogenesis level within a bone grafting area, immunohistochemical reaction with α-SMA antibodies (Abcam, USA) was performed, and the average number of vessels in 10 random fields of view was calculated. All specimens were scanned in the Mirax Scanner (Carl Zeiss, Germany); digital images were generated and evaluated qualitatively and quantitatively using Panoramic Viewer (3D Histech Ltd, USA). Using ImageJ (National Institutes of Health, USA), the areas of newly formed bone tissue were segmented to calculate their percentage in the total square of the defect (Supplementary Figure 2); this parameter was defined as the bone tissue formation rate.

## Clinical Trial

### Study Design

We conducted an open-label non-randomized clinical trial sponsored by Histograft LLC in the A. I. Evdokimov Moscow State University of Medicine and Dentistry (Moscow, Russia) from March 6, 2017 to December 14, 2018, according to the CONSORT guidelines (Schulz et al., 2010) excluding randomization. The clinical study protocol was approved by the Ethics Council of the Ministry of Health of the Russian Federation (approval No. 2 dated on 29.12.2016) and was published at ClinicalTrials.gov before beginning the trial (NCT03076138). The clinical trial was approved by the Federal Service for Surveillance in Healthcare (approval No. 610/2016 dated on 05.07.2016).

Patients aged 18–60 years with congenital and acquired maxillofacial bone defects (e.g., sockets of extracted teeth and bone defects after injuries, surgeries, removal of benign neoplasms, and pseudotumors) and alveolar ridge atrophy were enrolled into the trial after they signed the informed consent. The exclusion criteria were as follows: disability or unwillingness to give voluntary informed consent or to follow the requirements of the clinical trial; decompensated chronic diseases; clinically significant laboratory abnormalities; serum antibodies against human immunodeficiency virus, hepatitis B virus, and hepatitis C virus; alcohol consumption within 4 days prior to the study; a history of drug addiction; participation in other clinical trials (or the administration of investigated products) within 3 months prior to the study; conditions limiting study compliance (e.g., dementia, psycho-neurological diseases, drug addiction, and alcoholism); pregnancy or lactation; and malignancies including a post-treatment period (i.e., surgery, chemotherapy, radiation therapy both alone and in different combinations) <5 years prior to the study.

Upon enrollment, all patients underwent screening that included a set of instrumental investigations and laboratory tests as well as physical examination, and dental CT of the maxillofacial area, with assessment of baseline parameters of the bone defects or atrophy regions.

All patients enrolled in the study underwent bone grafting of the upper or lower jaw with the investigated gene-activated bone substitute (OCP/pDNA-*VEGF*) during a standard intervention (e.g., sinus-lifting procedure, cystectomy). Soft tissue suturing fixed the investigated material within the bone grafting area. In the postoperative period, all patients received standard medication therapy including antibiotics, analgesics, and desensitizing agents for 1–5 days. Antiseptic treatment of the oral cavity was performed at least three times a day until the sutures were removed at 8–10 days after surgery.

The clinical study results were evaluated at 1, 2, 10, 30, 90, and 180 days with clinical, laboratory, and instrumental investigations and tests. A control CT with the assessment of primary trial endpoints was conducted 180 days after surgery.

#### Outcomes

The primary trial endpoint was bone-tissue formation in the field of gene-activated bone-substitute implantation (bone grafting area). To quantify the newly formed bone tissue, the following morphometric parameters were measured on CT scans using standard tools: average density (in HU), size (length, width, and height in mm), and volume (in cm^3^). The average density was calculated as the total volume of newly formed bone tissues after preliminary segmentation in the 3DSlicer software (Brigham and Women's Hospital, Inc., USA). The treatment was considered successful when the tissue density in the bone grafting zone was within the range of the “bone window” (+450 to +1,500 HU) with a standard deviation not more than 20% of the mean value. The medical device was considered effective if the successful treatment rate was 90%.

Secondary endpoints included adverse events and serious adverse events: a surgical failure rate and a frequency of events when bone grafting could not be completed due to any reasons related to OCP/pDNA-*VEGF*.

In addition, for quantitative evaluation of specific characteristics of the post-surgical period, the principal investigator assessed the local pain level using the Visual Analog Scale, and edema was scored using the Numeric Rating Scale within the first 10 days after surgery.

#### Follow-Up

Patient clinical data (clinical examination, control CT scans to assess the stability of bone around the dental implants located in the bone grafted area) are still being collected, although the clinical trial has ended. At 6–8 months after surgery (within 2 months after completion of the clinical trial), all patients had dental implants placed in the bone grafting area, which allowed trephine biopsy sampling with a diameter of 2 mm and a length of 5–10 mm without a risk to the patients. The procedure was performed after the patients signed informed consent. Tissue specimens were prepared from samples fixed in 10% neutral formalin, using a standard procedure with Mallory trichrome staining. The specimens were then scanned in the Mirax Scanner (Carl Zeiss, Germany), and digital images were evaluated using Panoramic Viewer (3D Histech Ltd, USA). We then analyzed the presence of inflammatory infiltration, signs of gene-activated bone substitute bioresorption, bone formation, the interface between newly formed bone tissue, and the investigated material fragments.

## Statistical Analysis

### Experimental Studies

During descriptive statistical analysis, we calculated the mean, median, standard deviation, and the lower and upper quartiles. Given a non-normal distribution, the Mann-Whitney U-test was used to compare independent groups, with the Wilcoxon signed-rank test for intra-group comparisons at different time points. A *p* value < 0.05 was considered statistically significant.

### Clinical Trial

Given the clinical trial type, the sample size was calculated by determining the number of patients when a treatment success rate would be both clinically and statistically significant upon applying a method of confidence intervals to compare the frequencies of binary data in one group. The achievement of treatment success using the medical device in 90% of the cases was chosen as the threshold value of clinical effectiveness. In this case, the minimum sample size that provided statistical significance of differences between the treatment success (90%) and failure (10%) rates, calculated using the method of confidence intervals at a statistical significance level of 0.05, was 10 patients. To enhance the significance of statistical analysis, the calculated minimum sample size was doubled.

To describe quantitative data (i.e., the height of the maxillary alveolar process at the level of atrophy before and after bone grafting; the length, height, and width of the bone defect in the largest measurements before and after bone grafting; and the tissue density in the bone grafting area), the mean value (M), median (Me), standard deviation (σ), inter-quartile range (IQR), lower quartile (LQ), and upper quartile (UQ) were calculated. The distribution of quantitative parameters was evaluated using the Shapiro–Wilk test.

To compare the success and failure rates of bone grafting using the investigated medical device (rates of regeneration/failure to form in the medical device implantation zone in the range from +450 HU to +1,500 HU with a standard deviation of no more than 20% of the mean value), confidence intervals of relative rates were compared.

In addition, to characterize the bone grafting outcomes in detail and to evaluate the reproducibility of results for different groups of indications, additional statistical analysis of morphometric data was performed separately with two subgroups: patients with alveolar ridge atrophy and patients with jaw bone defects. The Wilcoxon (signed-rank) test was applied to compare the bone defect sizes before bone grafting and 6 months afterwards as well as the height of the maxillary alveolar ridge before bone grafting and 6 months later. The statistical significance of differences in the average density of newly formed tissues between subgroups was evaluated using the Mann-Whitney U-test. A significance level (*p*) of 0.05 was considered in all cases.

## Results

### *In vitro* Biocompatibility

**Figure 1:**
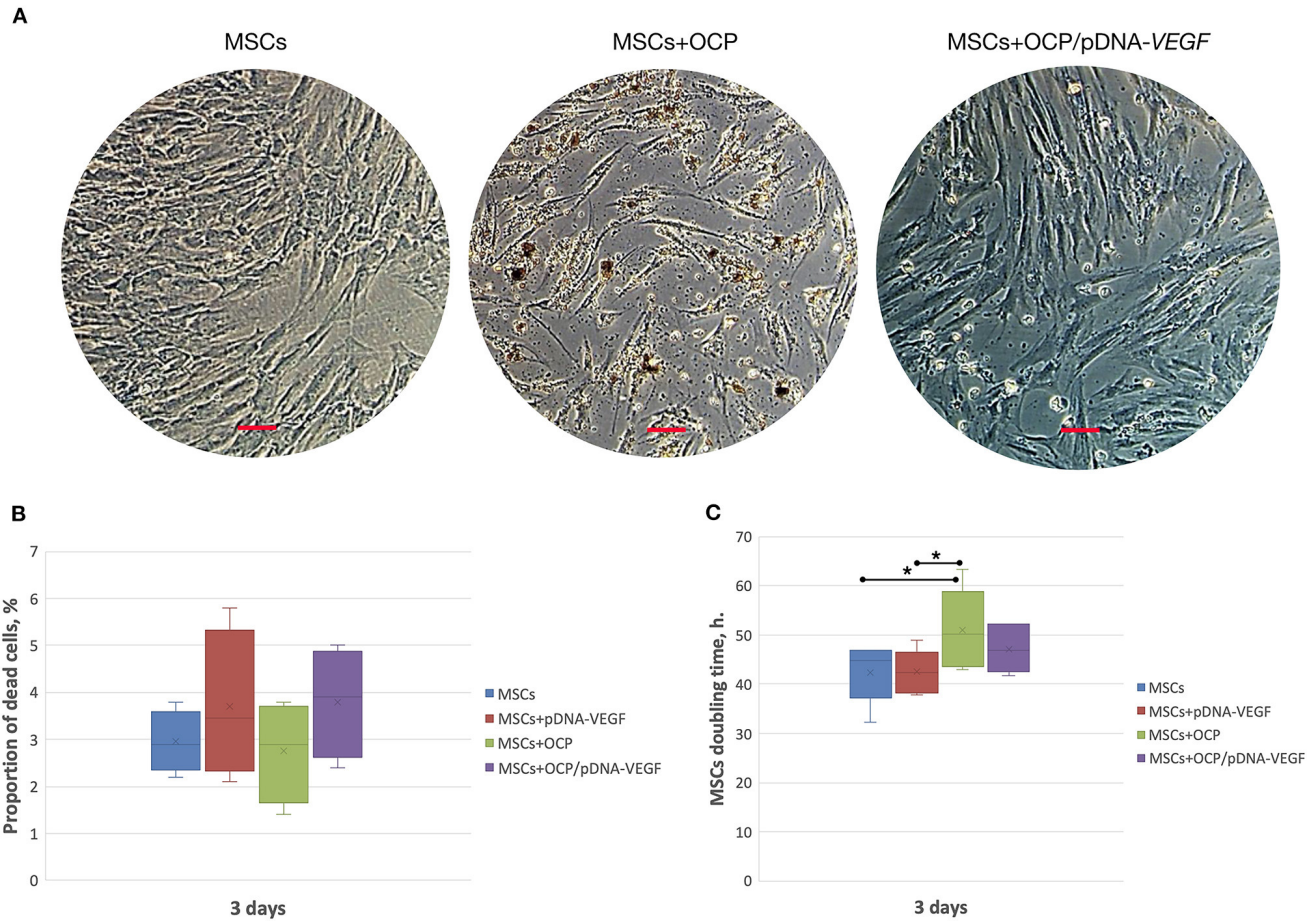
*In vitro* biocompatibility assay. **(A)** MSC-cultures, 3 days of co-incubation with the investigated materials; **(B)** number of dead cells; **(C)** cell culture doubling time. Scale bar: 50 μm. The asterisks above the lines connecting the groups indicate statistically significant differences (*p* < 0.05).

During co-incubation, OCP microparticles with diameters <3 μm were released from the investigated materials, passed through 3 μm-pores of Transwell inserts and reached the MSC monolayer. Some of the “microcrystallites” were detected inside the viable cells. Despite this finding, the average number of dead cells was similar in all groups and did not exceed the parameter of OCP-free cell cultures (*p* > 0.05). The MSC doubling time increased in the OCP group compared to that in the other controls, whereas this parameter remained unchanged in the materials-free (intact) MSCs, with a solution of pDNA-*VEGF* and OCP/pDNA-*VEGF* (*p* > 0.05) (Figure 1).

### *In vivo* Transfection Efficacy

**Figure 2:**
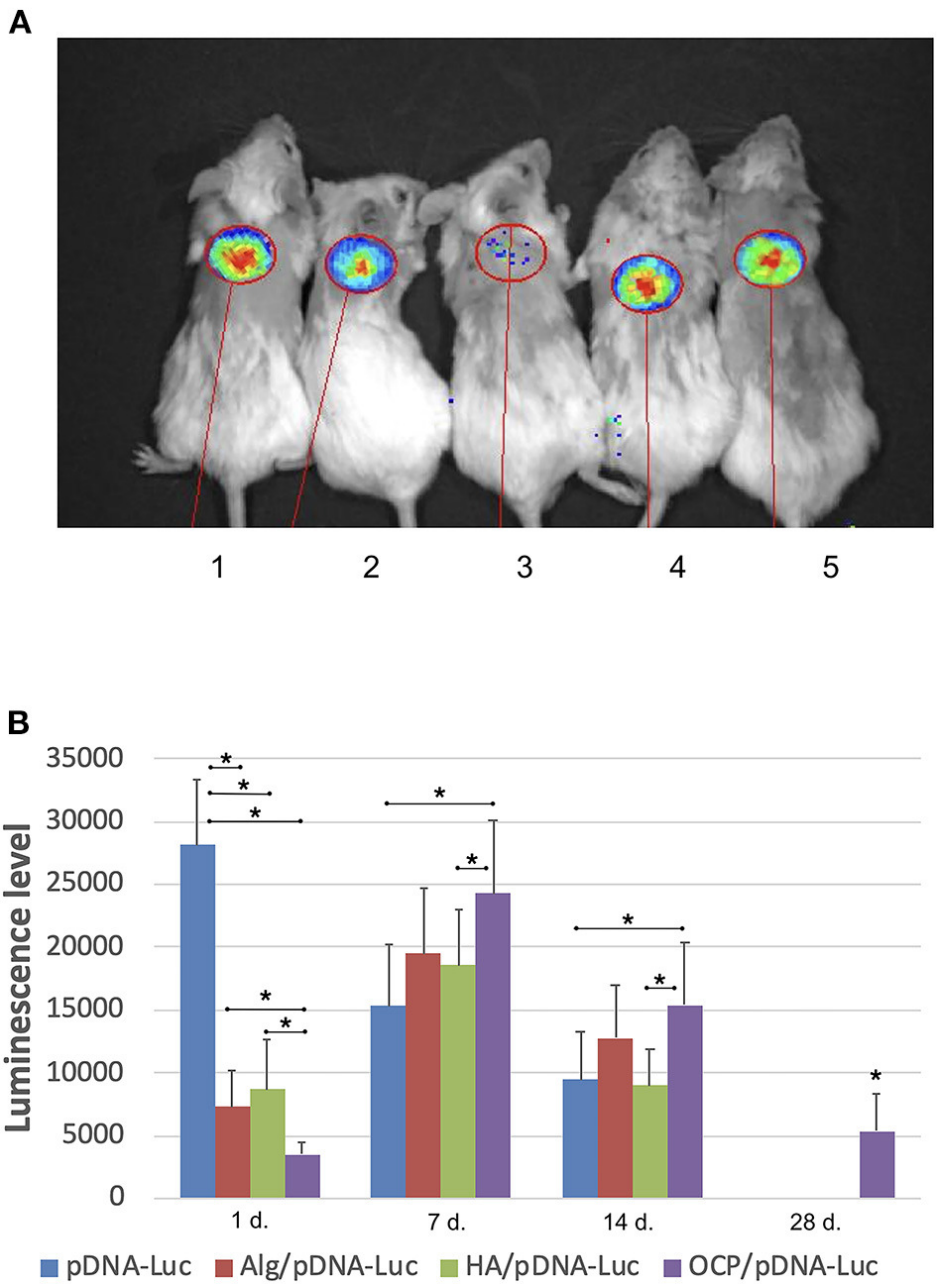
Plasmid DNA transfection efficacy *in vivo*. **(A)** Luminescence detection, day 7: 1 – OCP with *Luc*-carrying plasmid DNA, 2 –solution of plasmid DNA with *Luc* gene, 3 – OCP without plasmid DNA, 4 – alginate hydrogel with *Luc*-carrying plasmid DNA, 5 – hyaluronic acid hydrogel with *Luc*-carrying plasmid DNA; **(B)** dynamics of the luminescence level (p/s/cm^2^/sr). Vital luminescence bioimaging. The asterisks above the lines connecting the groups indicate statistically significant differences (*p* < 0.05). In the groups of OCP and pDNA-VEGF, only background luminescence was detected.

A luminescence signal was detected locally within the implantation area in all groups with pDNA-*Luc-*containing materials and solution. The maximal luminescence level was identified on day 1 after injecting the pDNA-*Luc* solution, and then, the signal gradually decreased and completely disappeared by day 28. In test group 1, the luminescence generated by OCP/pDNA-*Luc* was minimal at the first time point, peaked on day 7, slightly decreased by day 14, and was subsequently reduced by day 28. Alginate and hyaluronic acid hydrogels delivered pDNA-*Luc* in a similar manner: the initial level was less compared to the positive control group, then increased strongly, remained lower than that of OCP/pDNA-*Luc*, but dramatically declined to a zero level by day 28 (Figure 2).

### Bone Regeneration in the Cranial Model

None of the animals died during the experiment prior to the planned sacrifice, and the surgical wounds healed normally.

**Figure 3:**
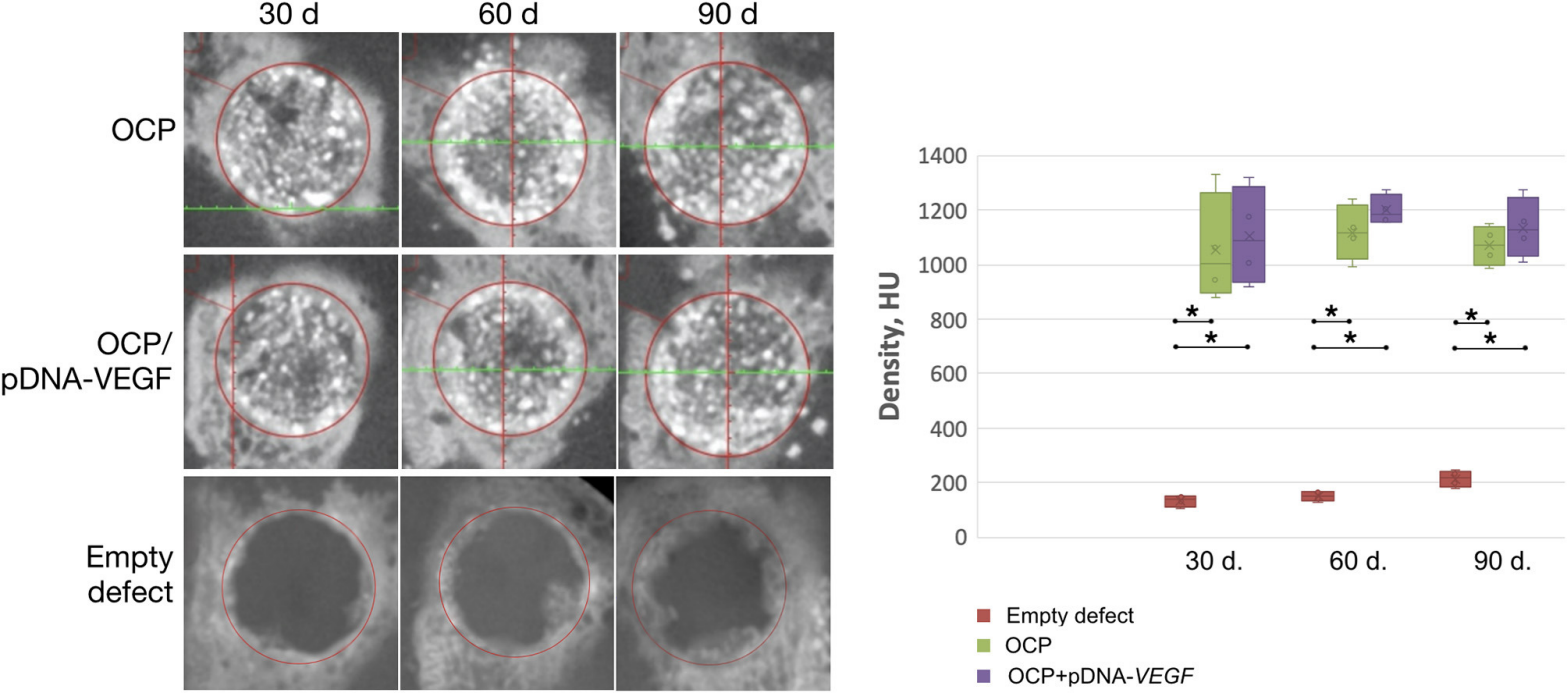
Critically sized bone defect repair in rabbits. CT data of parietal bones with circular defect segmentation. The asterisks above the lines connecting the groups indicate statistically significant differences (*p* < 0.05).

According to the CT data (Figure 3), at 30 days, the bone defect margins in all groups were well defined and were filled with granules of the implanted materials with a HU density similar to the surrounding intact cortical bone in the OCP/pDNA-*VEGF* and OCP groups. Regions with low density were detected between these granules. Within 60 days, the bone defect margins were smoothed and characterized by irregular shapes partially confluent with the peripherally located granules of the investigated materials. At 90 days, the region of the previously made defect in groups with bone grafting was identified only by accumulation of the partially resorbed OCP granules integrated into the newly formed tissues with high density. Meanwhile, in the control with blood clot, the complete defect was filled with low-density tissues on day 30. Later, some higher density tissues were formed from the defect margins, making them less distinguished, but the remaining defect of 7–8 mm in diameter was still retained by day 90 (Figure 3).

**Figure 4:**
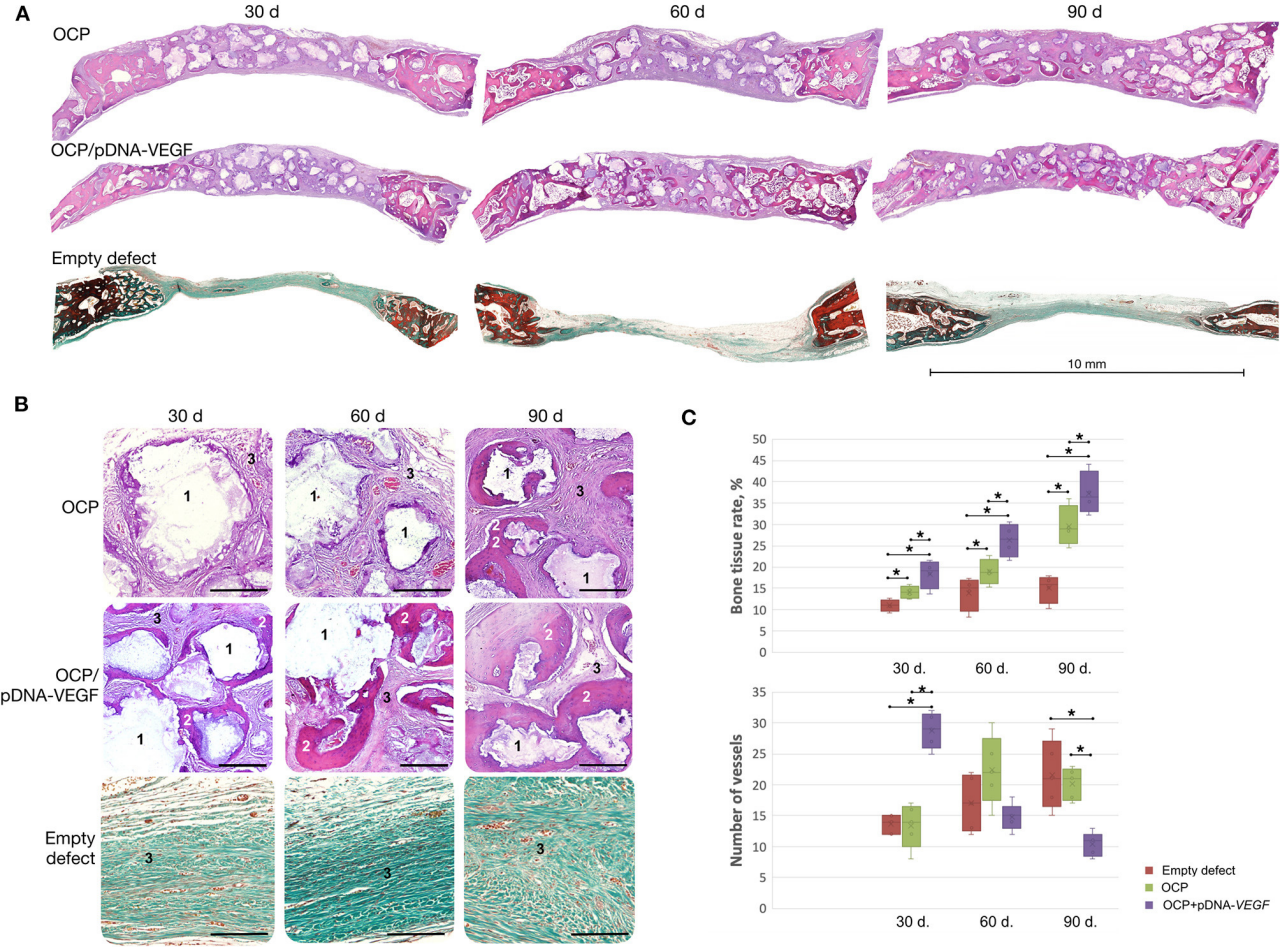
Critically sized bone defect repair in rabbits. **(A)** Full-defect histological images; **(B)** histological images from the central part of the defects at higher magnification: 1 – remaining fragments of implanted materials, 2 – newly formed bone tissue, 3 – fibrous tissue, H&E staining, scale bar: 300 μm; **(C)** quantitative evaluation of angiogenesis and bone formation; bone tissue rate is defined as the percentage of newly formed bone tissue in the total square of the defect (normal rate for the rabbit parietal bone measured with this method – 53 ± 3.5%). Bone defects were mostly filled by fibrous tissue in the empty defect group confirming the model to be critical-sized. The asterisks above the lines connecting the groups indicate statistically significant differences (*p* < 0.05).

Based on histological analysis (Figure 4), woven bone tissue was formed from the defect margins directly involving the nearest fragments of OCP or OCP/pDNA-*VEGF*. These granules were in direct contact with the newly formed bone without fibrous tissue between guided bone growth and the central part of the defect. However, only in the OCP/pDNA-*VEGF* group, bone tissue formation was detected around the gene-activated material fragments located in the central part of the defect, i.e., distant from the defect margins, from day 30. By day 90, newly formed bone tissue was observed throughout the previously made bone defects in both the OCP and OCP/pDNA-*VEGF* groups, but in the latter, the bone tissue formation rate was significantly higher at all time points (Figure 4). Additionally, there were some bone marrow formation sites in the OCP/pDNA-*VEGF* group on days 60 and 90, whereas OCP without pDNA-*VEGF* did not facilitate such a process (Supplementary Figure 3). In the control with blood clots, osteogenesis activity decreased by days 60–90, which resulted in a fibrous scar occupying ~7 mm of the defect diameter (Figure 4).

The number of vessels was found to be significantly higher in OCP/pDNA-*VEGF* at the earliest time point. Later, the parameter gradually decreased to a minimal level on day 90, whereas it increased in the OCP group, reaching almost equal values by day 60 and finally became significantly higher (Figure 4C).

### Clinical Study Results

#### Patients

The group comprised 20 patients (6 men and 14 women) with an average age of 46.6 ± 11.9 years, having the most common dental and maxillofacial indications for bone grafting such as alveolar ridge atrophy, chronic periodontitis, radicular cysts, and peri-implantitis (Table 1). All patients planned to undergo specialized implant treatment for dental implant-based prosthetics. Surgical intervention using the investigated medical device was feasible in all cases, and the surgical failure rate was 0%. On average, 1.5 ± 0.5 g (≅1.5 ± 0.5 cm^3^) of the medical device was used for treating each patient.

**Table 1:** Characteristics of patients enrolled in the clinical trial.

**Characteristics of study population**	**Number of patients (*n* = 20)**
**Gender**	
Male, *n* (%)	6 (30 %)
Female, *n* (%)	14 (70 %)
**Mean age**, *M* ±σ years	46.6 ± 11.9
**Diagnosis**, ***n***	
*Atrophy of alveolar ridge*	**16**
Unilateral atrophy of the maxillary alveolar process (left or right)	11
Bilateral atrophy of the maxillary alveolar process	5
*Bone defects*	**4**
Chronic periodontitis of one or more teeth	2
Radicular cyst with the causative tooth	1
Peri-implantitis	1

Nineteen patients had all six visits as planned in the clinical trial protocol with the last visit at 6 months after surgery. One patient completed the trial 1.5 months before the estimated date at his own request. His data from the last visit (4.5 months) were used in the analysis of the study results along with the other patient data obtained at 6 months after surgery.

#### Safety

No adverse events or serious adverse events were recorded during the clinical trial. In all patients, the postoperative wounds healed by primary intention without abnormalities, and the sutures were removed at 8–10 days after surgery (visit free). The mean postoperative pain score peaked on postoperative day 2 at 3.95 ± 0.94, and was controlled using pain relievers (Ketanov, 10 mg per os, 2 times per day for 2–3 days). By day 10, pain resolved completely in all patients and no pain relief was required thereafter. Later, none of the patients reported any tenderness or discomfort within the postsurgical area. The average edema score was 4.7 ± 1.08, as rated by the Numeric Rating Scale on postoperative day 2. On day 10 after surgery, no swelling was observed.

There were no clinically significant abnormalities in the results of physical examination, blood tests, urinalysis, or electrocardiogram at all time points. Eight patients had single clinically insignificant abnormalities in the blood test or urinalysis without any progression (Supplementary Table 1). There were no radiographic or physical examination findings of inflammation or excessive hyperplasia of the maxillary sinus mucosa in patients who underwent sinus lifting with the investigated medical device.

#### Efficacy

**Figure 5:**
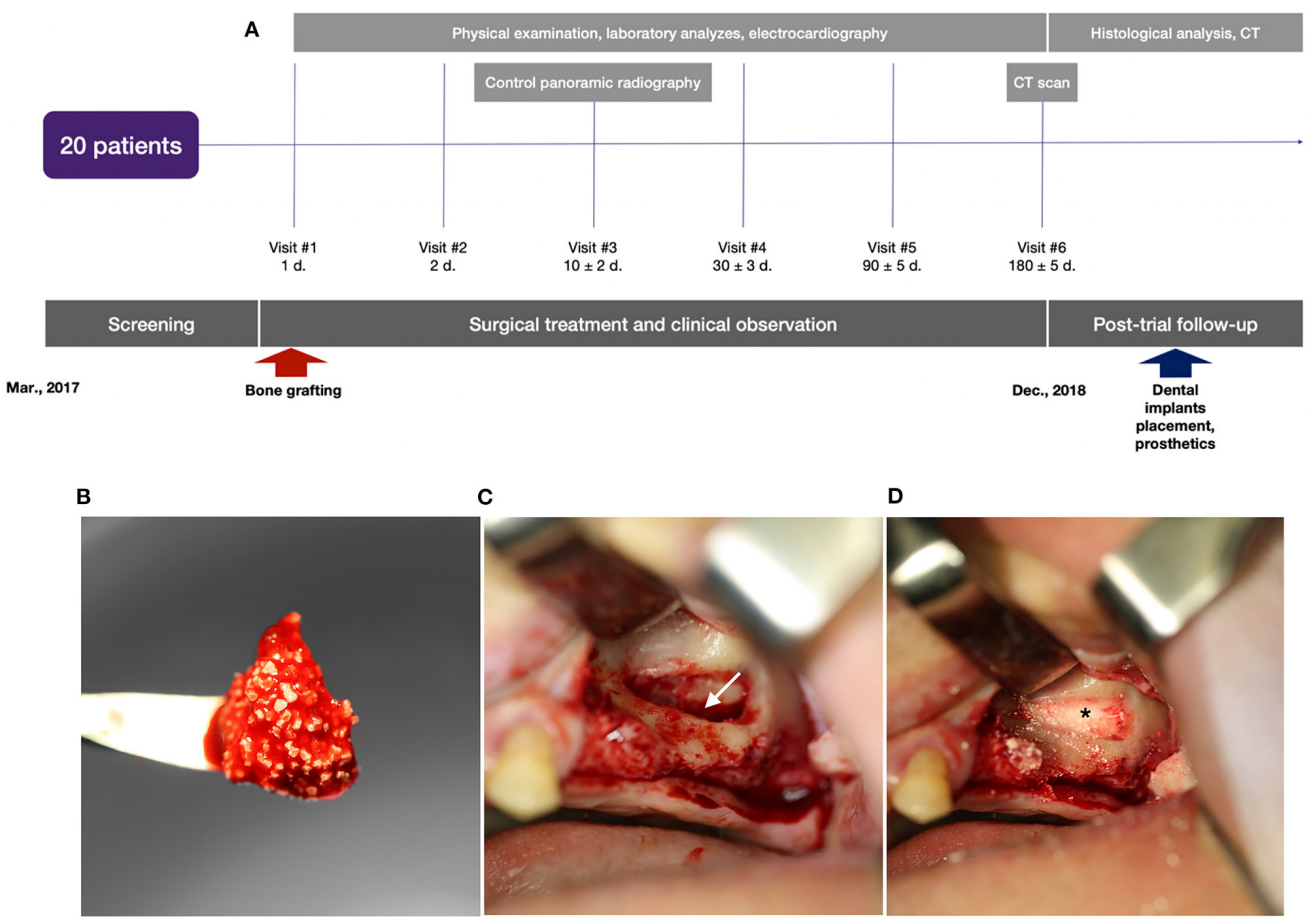
Clinical trial with illustration of the typical indication for bone grafting: **(A)** scheme of the clinical trial design, **(B)** granules of the gene-activated bone substitute in the blood clot before implantation into the bone defect; **(C)** the space (indicated by the white arrow) opened to be filled in by the gene-activated bone substitute in the sinus-lifting procedure; **(D)** granules of the implanted gene-activated bone substitute (indicated by the asterisk) within the bone cavity.

Implantation of the gene-activated bone substitute was technically feasible and simple (Figure 5). Granules of the medical device were easily wettable with a 0.9% sodium chloride solution or the patient's blood, and formed aggregates (Figure 5B) suitable for placement and fixing in the bone grafting zone.

Qualitative analysis of the radiographic results showed that the investigated gene-activated bone substitute was strictly within the zone of implantation without any signs of dislocation within 10 days after bone grafting. Six months after the intervention, newly formed tissues were identified within the bone grafting site of all patients; these tissues corresponded to the bone tissue in density (Table 2), and their volume corresponded with the amount of medical device granules implanted.

**Table 2:** Mean density values of newly formed tissues in 6 months after implanting the gene-activated bone substitute.

**Patient number**	**Density of newly formed bone tissue, HU (M ± σ)**	**Outcome, +/–**
1	887.8 ± 172.43	+
2	*1, 119.68* ± 201.19	+
3	863.22 ± 111.49	+
4	*1, 009.37* ± 174.10	+
5	948.59 ± 121.88	+
6	981.56 ± 150.88	+
7	*1, 067.13* ± 210.46	+
8	902.45 ± 105.35	+
9	774.56 ± 118.30	+
10	817.85 ± 135.51	+
11	716.37 ± 140.22	+
12	*1, 100.02* ± 147.99	+
13	912.13 ± 181.36	+
14	855.45 ± 162.93	+
15	864.71 ± 115.80	+
16	715.7 ± 99.57	+
17	951.27 ± 128.31	+
18	991.2 ± 144.53	+
19	836.82 ± 129.58	+
20^*^	846.81 ± 192.71	+
**Mean value**	**908.13 ± 114.40**	
**Total, success rate, %**		**100**

* *Data from the patient who completed the clinical trial at 4.5 months after surgery*.

**Figure 6:**
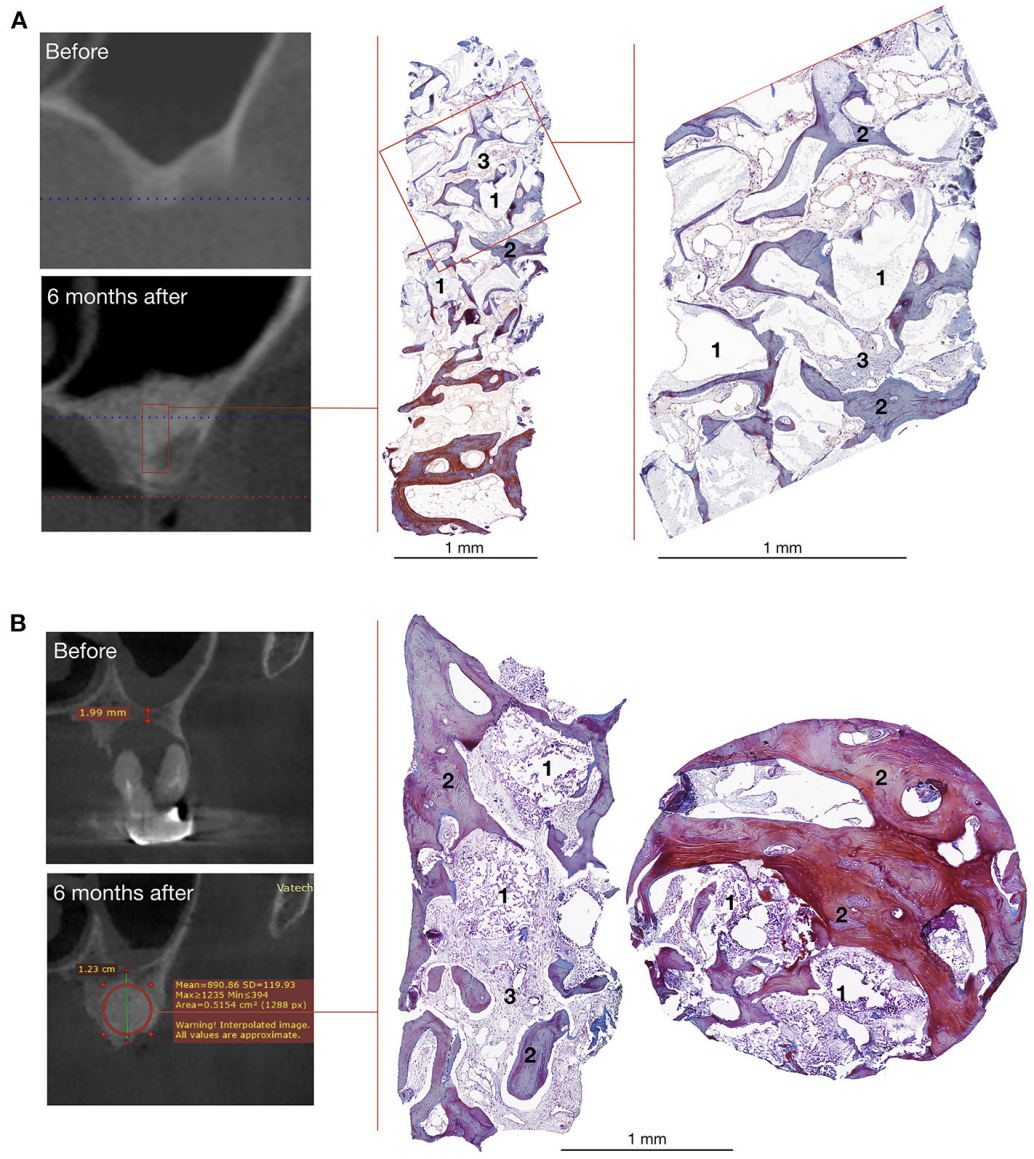
Bone grafting results in patients with unilateral alveolar ridge atrophy **(A)** and bone defect caused by radicular cyst **(B)**. On the left – CT scans, on the right – histological images of the trephine biopsies: 1 – gene-activated bone substitute fragments, 2 – newly formed bone tissue, 3 – fibrous tissue, Mallory trichrome staining.

**Figure 7:**
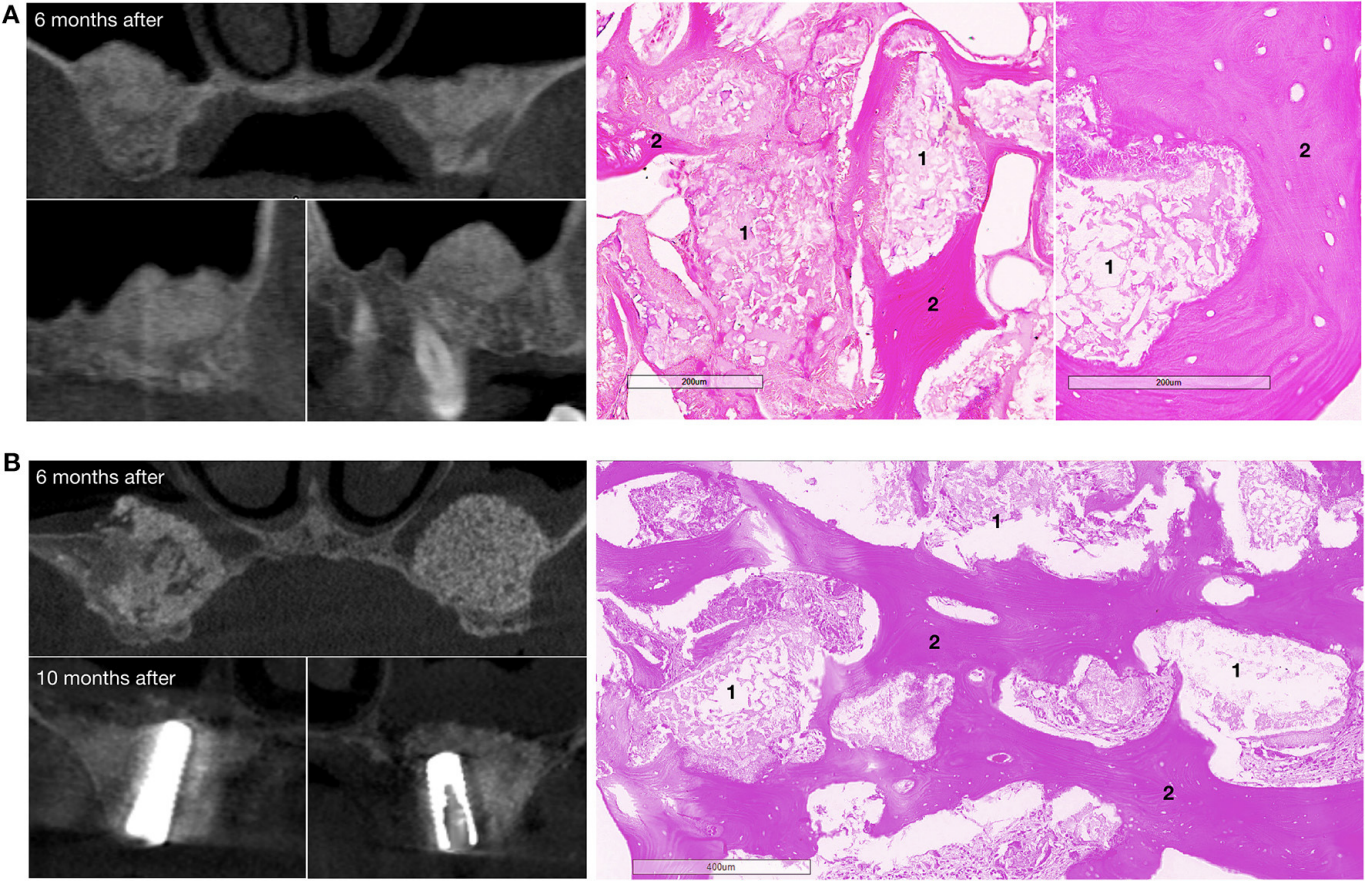
Bone grafting results in patients with bilateral alveolar ridge atrophy. **(A)** The patient completed the clinical trial, on the left – CT scans: upper image – coronal view, bottom images – sagittal view, on the right – histological images of the trephine biopsy; **(B)** another patient completed the clinical trial, on the left – CT scans: upper image – coronal view, 6 months after surgery, bottom images – coronal view, 10 months after surgery and 4 months after dental implant placement; on the right – histological images of the trephine biopsy. 1 – gene-activated bone substitute fragments, 2 – newly formed bone tissue. H&E staining.

CT scans demonstrated that bone grafting using the investigated medical device was successful in all patients, with a successful bone grafting rate of 100% at 6 months after surgery. The density ranged within the bone window from +450 to +1,500 HU with a standard deviation of <20%. The average bone density was 908.13 ± 114.40 HU (normal distribution, *p* = 0.778) within the site of bone grafting (Table 2, Figures 6, 7).

The mean newly formed tissue density value was 915.28 ± 125.85 HU and 879.56 ± 48.36 HU (*p* = 0.603) in the subgroups of patients with maxillary alveolar process atrophy and bone defects, respectively. At 6 months after bone grafting, the average height of the maxillary alveolar process in the zone of previously diagnosed atrophy was increased by 8.86 ± 4.33 mm (864.02 ± 469.82%) and reached 12.27 ± 1.96 mm, compared with the baseline value of 1.64 ± 0.92 (*p* < 0.001).

The baseline volume of bone defects in the subgroup of patients with cysts, peri-implantitis, and chronic apical periodontitis was as follows: Me = 872.76 mm^3^; *M* = 1,260.12 mm^3^; IQR = 2,656.13 mm^3^; LQ = 679.16 mm^3^, and UQ = 3,335.28 mm^3^ (non-normal distribution, *p* = 0.009). Six months after implanting the gene-activated bone substitute, complete bone healing was observed in 100% of the cases, with no residual bone defects identified.

#### Post-trial Follow-Up

At the time of writing, all patients who completed the clinical trial were followed up; the follow-up time of the first enrolled patient was 30 months. Neither adverse events nor serious adverse events were recorded in any patient. All patients had dental implants and prosthetics placed with an optimal primary stability. All patients signed an informed consent form before collecting trephine biopsy samples during dental implantation.

Histological examination showed that within 6–8 months after surgery, newly formed bone tissue occupied most of the area in all cases, spreading from the recipient bone to the outlying zone (Figures 6, 7). Partially resorbed granules of the gene-activated bone substitute were entirely integrated with the newly formed bone tissue without any connective tissue between them.

## Discussion

To date, bone reconstruction procedures are challenging in traumatology and orthopedics as well as in oral and maxillofacial surgery because effective alternatives to bone autografts are scarce and have only restricted indications for use. For example, a recombinant BMP-2-containing activated substitute was indicated for spinal fusion (Burkus et al., 2002) and alveolar ridge augmentation (McKay et al., 2007).

We have previously demonstrated that an OCP-based bone substitute could improve reparative osteogenesis *in vivo* (Komlev et al., 2014; Bozo et al., 2020). However, a lack of osteoinductive capacities has been observed in experimental studies, which could explain why a significant part of the newly formed tissues in the biopsy samples was presented by fibrous tissue in the clinical study. In addition, we participated in the development of Neovasculgen (HSCI, Russia), a gene therapy drug that is effective for treating patients with chronic lower limb ischemia, and has been approved for clinical use since 2011 (Deev et al., 2018). Given the critical role of angiogenesis for bone regeneration in embryonic and postnatal ontogenesis, as well as our own experience with gene therapy and bone grafting, and the above-mentioned drawbacks of OCP ceramic we developed a bone substitute based on OCP and plasmid DNA carrying the *VEGFA* gene.

Until recently, gene-activated bone substitutes formed a “scaffold-related part” of gene therapy that is promising in replacing bone autografts (D'Mello et al., 2017), and have had no precedent of clinical translation. A low delivery level of gene-based constructs into cells *in vivo*, especially of naked plasmid DNA, is the main challenge for this category of activated bone substitutes. However, we managed to bypass this using OCP as a scaffold. It has been previously assumed that calcium phosphate materials can enhance plasmid DNA delivery through mechanisms similar to calcium phosphate-mediated optimization of transfection *in vitro* (Keeney et al., 2010). We obtained some findings supporting this hypothesis. In an *in vitro* experiment, we found that OCP granules release microparticles with diameters <3 μm that are accepted by cells, and can theoretically carry plasmid DNA molecules. However, additional research should be conducted to prove this assumption. Simultaneously, the levels of *VEGFA* mRNA and VEGF-A165 protein secretion *in vitro* were significantly higher even without the use of transfection agents when a gene-activated bone substitute rather than a plasmid DNA solution was used. In particular, the level of *VEGFA* mRNA expressed in MSCs co-incubated with OCP/pDNA-*VEGFA* peaked at day 5, reaching 45.0 ± 3.9, whereas in the groups of material-free culture and pDNA-*VEGFA*, the mRNA level was 7.6 ± 2.1 and 4.4 ± 1.2, respectively. OCP without plasmid DNA increased the mRNA expression of *VEGFA* to 19.2 ± 7.9. At day 5, the accumulated concentration of VEGF-A165 (the culture medium was not changed during the experiment) was 1,625.3 ± 185.3 pg/ml in the OCP/pDNA-*VEGFA* group, whereas the material-free MSC culture secreted 1,466.1 ± 136.7 pg/ml, and pDNA-*VEGFA* and OCP groups showed concentrations of 1,412.6 ± 114.5 pg/ml and 1,295.5 ± 37.2 pg/ml, respectively (Bozo et al., 2016).

Similar data were obtained in a subcutaneous bioluminescence test using the *Luc*-carrying marker DNA construct. pDNA-*Luc* delivery in an aqueous solution (positive control) reached a peak level much quicker (on day 1), most likely because all the molecules were available for transfection immediately after administration. pDNA release from hyaluronic acid and alginate hydrogels was slower, but optimal kinetics with the highest and longest transgene expression were observed in the OCP/pDNA-*Luc* group, providing luciferase production for at least 28 days. Although the results obtained from *in vivo* experiments with the reporter plasmid cannot be directly extrapolated to VEGF expression, these data proved that the OCP scaffold could deliver plasmid DNA *in vivo*, and that the release of the gene constructs was more prolonged compared to a solution or some hydrogels. Together with the *in vitro* data on VEGF expression (Bozo et al., 2016), preclinical studies have suggested that the investigated gene-activated bone substitute can increase VEGF expression.

An experimental study of healing in critically sized cranial bone defects in rabbits showed that in the early stages after surgery, OCP/pDNA-*VEGF* enhanced angiogenesis and provided bone tissue formation from intact bone surfaces as well as in the central zone, without any contact with the native bone tissue, where only OCP/pDNA-*VEGF* could induce bone formation. The decreasing trend in the number of vessels in the OCP/pDNA-*VEGF* group and the opposite in the OCP group from 30 to 90 days could be explained by the differences in microvasculature between bone and fibrous scar tissues as the latter has a larger serpentine network of vessels that increases their density on the histological slice. This effect of the investigated material could be proposed as evidence of osteoinduction that in combination with successful results of technical and toxicology studies and manufacturing (data not shown), led us to initiate clinical trial as part of the medical device registration in Russia.

It is generally recognized that randomized controlled clinical trials are more evidence-based (Bhatt and Mehta, 2016). However, the Russian legislation allows researchers to conduct studies in the form of an open-label non-randomized clinical trial, especially if there is no common recognized standard for comparison, and the effectiveness parameters (primary outcome) for medical devices are well-defined, generally accepted, and can be measured and analyzed without comparison with the control. Bone substitutes indicated for oral and maxillofacial surgery fall into this category for the following reasons. First, bone autografts in the form of blocks are not applicable for sinus-lifting procedures, the most common type of surgery for alveolar ridge augmentation, and bone autografts in the shredded form are not the “gold standard,” as they undergo rapid bioresorption. Therefore, it is recommended to mix them in various combinations with osteoconductive bone substitutes (Cordaro and Terheyden, 2015), of which more than 100 variants are approved for clinical practice. Second, the criteria for bone grafting success in the maxillofacial region are definitive and logical: obtaining the required volume of newly formed tissues that correspond to bone in density according to CT scans (mean value from +450 HU to +1,500 HU with a standard deviation <20%). Therefore, we conducted our clinical study as an open-label, non-randomized clinical trial. The last time point of the study, i.e., 6 months, coincides with the duration of new bone tissue formation and maturation, and is the average waiting period prior to dental implant placement.

The use of the investigated medical device was simple and comparable to the implantation of other granulated substitutes. Simultaneously, because of its hydrophilic surface, the gene-activated bone substitute could be wetted with a 0.9% NaCl solution and/or the patient's blood, facilitating and accelerating the process of placing granules into the area of bone grafting.

The use of the investigated medical device was safe, as there were no adverse events or severe adverse events, and the pain and edema levels corresponded to the intervention severity. Moreover, according to previously published “Neovasculgen” biodistribution studies, plasmid DNA administered intramuscularly at the dosage of 2.4 mg (24 times more than in 1.0 g of the gene-activated bone substitute) had a short-term and non-significant presence in blood without an increase in VEGF-A165 protein levels (Yudin et al., 2015). Based on these data, no systemic effect assessments of the investigated medical devices were required.

Bone grafting using the OCP/pDNA-*VEGF* was effective in 100% of the patients, as tissues corresponding to bone tissue in density were formed within the intervention area, with a mean density value of 908.13 ± 114.40 HU, which is covered by d2-type bone tissue under the Misch classification (Misch, 1990) or type 2 spongy bone under the classification of Lekholm and Zarb (1985). This bone type is optimal for placing dental implants, and providing adequate primary stability and steady osteoinduction (Makary et al., 2019). Notably, equally successful results were obtained in a subgroup of patients with atrophy and in one with bone defects; thus, we can reasonably expect the reproducibility of results for different indications in bone surgery; however, additional clinical studies aimed at specific indications are needed. The follow-up period, which has already been 30 months for the first patient, supports the safety and efficacy of the device because no patient has lost a dental implant, nor shown active resorption of the bone tissue within the peri-implant area to date.

Histological examination results present additional value as they are extremely rare in clinical studies on bone substitutes and can be obtained only when bone grafting is a preparation step for other surgical interventions for the purpose of reconstruction or rehabilitation. We identified newly formed tissues within the area of surgery to consist of regenerated bone and partially resorbed granules of the bone substitute integrated into them. Regions of connective tissue were minimal. This correlates with a low variance of the mean density value in each patient (standard deviation ≤ 20%) and supports the device effectiveness.

Thus, our findings suggest that using the developed gene-activated bone substitute could be efficacious for bone grafting in the maxillofacial area. However, only subsequent randomized controlled clinical trials will determine the maximal increase in the alveolar ridge that could be achieved in each specific group of indications for bone grafting procedures as well as the possibility of using this gene-activated bone substitute in traumatology and orthopedics for reconstructing large bone defects.

## Data Availability Statement

The raw data supporting the conclusions of this article will be made available by the authors, without undue reservation.

## Ethics Statement

The studies involving human participants were reviewed and approved by Ethics Council, Ministry of Health of the Russian Federation. The patients/participants provided their written informed consent to participate in this study. The animal study was reviewed and approved by Interuniversity Ethics Committee, Moscow, Russia.

## Author Contributions

IB, RD, and AI: preclinical and clinical studies conception. IB and RD: preclinical and clinical data interpretation. IB, RD, and VK: preclinical data collection and analysis. AD and NR: clinical trial conception, clinical data collection, and interpretation. IB: manuscript drafting. All authors critically revised the manuscript and gave final approval.

## Conflict of Interest

The clinical trial, as part of registering a gene-activated bone substitute for clinical use, was carried out under Russian Ministry of Healthcare regulations. Based on an official report examination performed by an expert government institution, market authorization was granted in 2019. The clinical trial and registration process were funded by Histograft LLC. IB, AI, and RD are co-owners of the company. In addition, IB and RD are Histograft employees who participated in the development of the clinical study design in cooperation with the principal investigator (AD); no CRO companies are required for bone substitute registration in the Russian Federation. IB, AI, and RD were not involved in the clinical data collection, analysis, or official report writing. For manuscript writing, all the results were transferred from a clinical trial official report submitted to the Ministry of Healthcare. Additional follow-up data were collected by AD and NR and were analyzed together with IB and RD. The investigated bone substitute is covered by a patent application “Methods of producing optimized gene-activated materials,” US 20190224379A1, patent applicant - Histograft, LLC; inventors: RD, AI, IB, and VK.

## Supplementary Material

Supplementary Figure 1 Expression map of pDNA-*VEGF* (length, 4859 base pairs). pDNA-*VEGF* contains elements that allow expression of a target gene in mammalian cells: the CMV early promoter/enhancer region (nucleotides 4626–355) including the enhancer region (nucleotides 4684–231) and cytomegalovirus promoter (nucleotides 274–355); the TATA box-element (nucleotides 320–326), transcription start (349); polyadenylation signal, and virus SV40 terminator (nucleotides 1306–1531); and elements that maintain the presence of the plasmid in bacterial cells, i.e., the region of initiation of bacteriophage replication f1 (nucleotides 1583–1995), prokaryotic promoter of the gene bla (nucleotides 2058–2086), sequence encoding aminoglycoside-3′-phosphotransferase providing bacterial resistance to kanamycin (nucleotides 2519–3313), and region of plasmid replication from pBR322 (nucleotides 3907–4526). The region between nucleotides 362–967 includes the Kozak sequence (nucleotides 380–391) located around the start codon of a target gene and provides the initiation of translation of mRNA of the target gene, the open reading frame of the gene encoding VEGF 165 (nucleotides 392–964), and the stop-codon (nucleotides 965–967). The plasmid contains recognition sites for restriction endonucleases NdeI (1), BamHI (364), EcoRI (373), BsrGI (854), XmaI (969), Acc651 (978), BclI (1307), NarI (2650), ApaLI (4254), and PciI (4568).

Supplementary Figure 2 Histological image with newly formed bone tissue segmented using ImageJ software.

Supplementary Figure 3 Bone marrow formation sites in the OCP/pDNA-*VEGF* group, 60 days after implantation. H&E staining.

Supplementary Table 1 Abnormalities detected in the blood test and urinalysis in patients participated in the clinical trial. MCH, mean corpuscular hemoglobin; HGB, hemoglobin; GLU, glucose; HCT, hematocrit; RBC, red blood cells; EOS, eosinophils; BIL-T, total bilirubin; hpf, high-power field. ^*^Normal values are presented according to the laboratory performed the analysis.
