# Supplementary figures and images for: Bringing a Gene-Activated Bone Substitute Into Clinical Practice: From Bench to Bedside
Source: Front Bioeng Biotechnol. 2021 Feb 4;9:599300. doi: 10.3389/fbioe.2021.599300 (PMC7889956; doi:10.3389/fbioe.2021.599300)

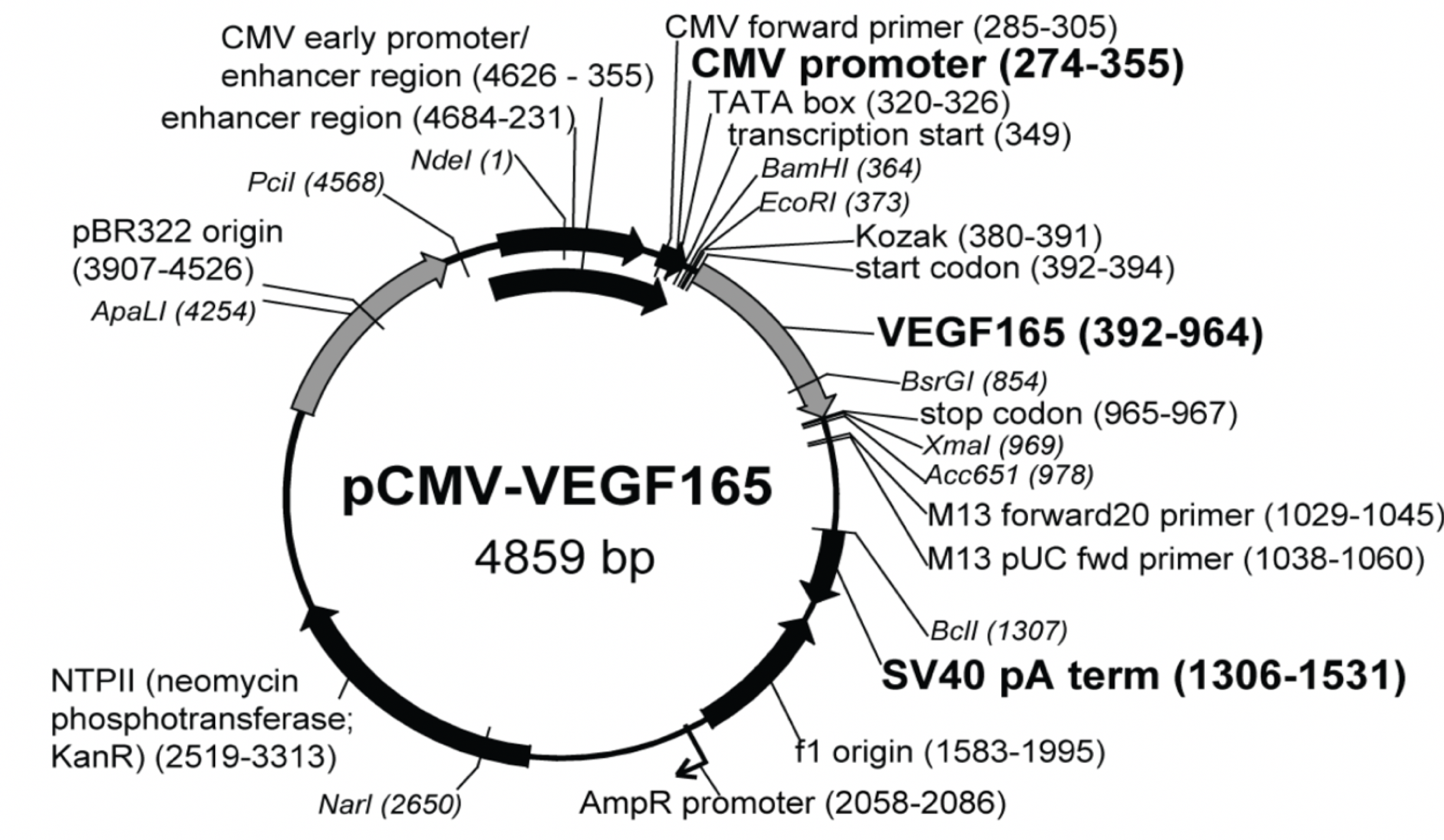

Supplement: Supplementary Figure 1 — Expression map of pDNA-VEGF (length, 4859 base pairs). pDNA-VEGF contains elements that allow expression of a target gene in mammalian cells: the CMV early promoter/enhancer region (nucleotides 4626–355) including the enhancer region (nucleotides 4684–231) and cytomegalovirus promoter (nucleotides 274–355); the TATA box-element (nucleotides 320–326), transcription start (349); polyadenylation signal, and virus SV40 terminator (nucleotides 1306–1531); and elements that maintain the presence of the plasmid in bacterial cells, i.e., the region of initiation of bacteriophage replication f1 (nucleotides 1583–1995), prokaryotic promoter of the gene bla (nucleotides 2058–2086), sequence encoding aminoglycoside-3′-phosphotransferase providing bacterial resistance to kanamycin (nucleotides 2519–3313), and region of plasmid replication from pBR322 (nucleotides 3907–4526). The region between nucleotides 362–967 includes the Kozak sequence (nucleotides 380–391) located around the start codon of a target gene and provides the initiation of translation of mRNA of the target gene, the open reading frame of the gene encoding VEGF 165 (nucleotides 392–964), and the stop-codon (nucleotides 965–967). The plasmid contains recognition sites for restriction endonucleases NdeI (1), BamHI (364), EcoRI (373), BsrGI (854), XmaI (969), Acc651 (978), BclI (1307), NarI (2650), ApaLI (4254), and PciI (4568). [file Image_1.TIF]

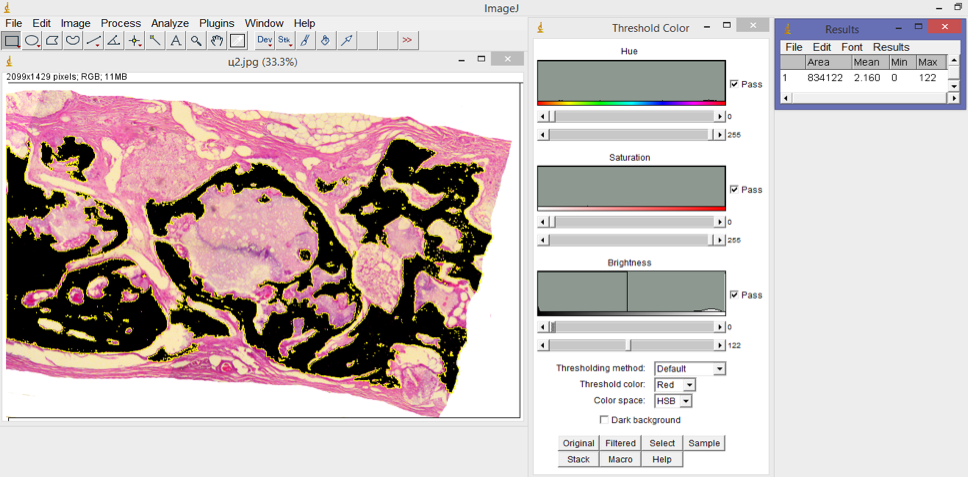

Supplement: Supplementary Figure 2 — Histological image with newly formed bone tissue segmented using ImageJ software. [file Image_2.TIF]

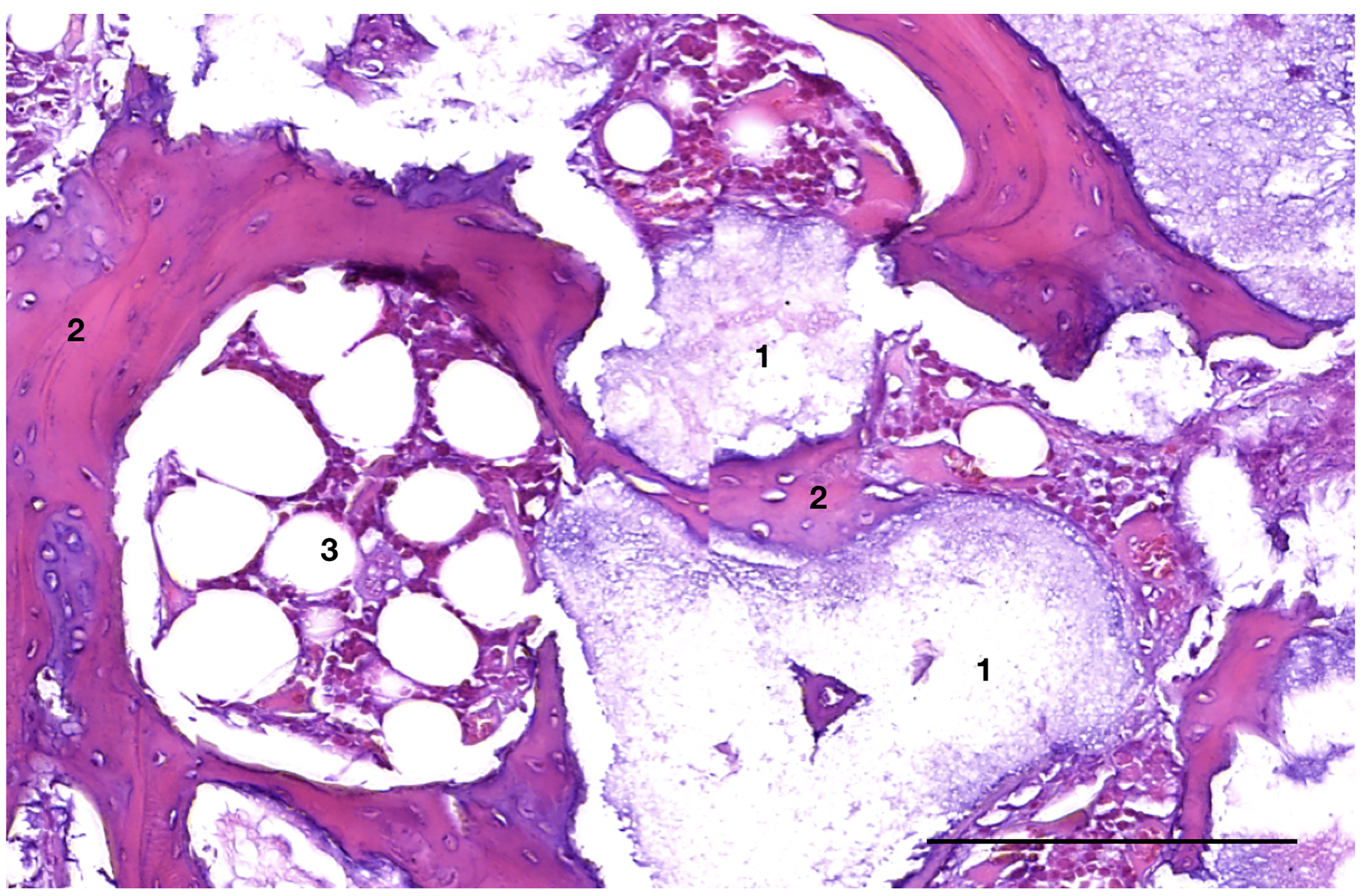

Supplement: Supplementary Figure 3 — Bone marrow formation sites in the OCP/pDNA-VEGF group, 60 days after implantation. H&E staining. [file Image_3.TIF]
