# Supplementary material for: Bringing a Gene-Activated Bone Substitute Into Clinical Practice: From Bench to Bedside
Source: Front Bioeng Biotechnol. 2021 Feb 4;9:599300. doi: 10.3389/fbioe.2021.599300 (PMC7889956; doi:10.3389/fbioe.2021.599300)
Supplement: Supplementary Table 1 — Abnormalities detected in the blood test and urinalysis in patients participated in the clinical trial. MCH, mean corpuscular hemoglobin; HGB, hemoglobin; GLU, glucose; HCT, hematocrit; RBC, red blood cells; EOS, eosinophils; BIL-T, total bilirubin; hpf, high-power field. *Normal values are presented according to the laboratory performed the analysis. [file Table_1.DOCX]

| **Patient number** | **Laboratory test abnormality / time point (d)** | **Normal values*** |
| --- | --- | --- |
| 3 | **Blood test**  MCH – 26.4 pg / 10 d | 27.0-34.0 pg |
| 4 | **Blood test**  HGB – 124 g/l / 10 d  Prothrombin time – 14.1 sec / 180 d | HGB 126-174 g/l  Prothrombin time 10.0-13.2 sec |
| 8 | **Blood test**  GLU – 6.3 mM/l / 10 d  **Urinalysis**  WBC – 6/hpf | GLU 4.1-6.0 mM/l  WBC 0-5/hpf |
| 11 | **Blood test**  HGB – 178 g/l / 180 d  HCT – 52.1 % / 180 d | HGB 126-174 g/l  HCT 37.0-51.0 % |
| 13 | **Urinalysis**  RBC - 3/hpf / 10 d | RBC 0-2/hpf |
| 15 | **Blood test**  EOS – 6.2 % | EOS 1.0-5.0 % |
| 17 | **Blood test**  HGB – 120 g/l / 10 d  **Urinalysis**  pH – 4.7/ 10 d  WBC – 7/hpf / 180 d | HGB 126-174 g/l  pH – 5.0-8.0  WBC 0-5/hpf |
| 20 | **Blood test**  BIL-T – 21.8 mM/l / 10 d. | BIL-T 3.4-20.5 |

**Supplement table.** Abnormalities detected in the blood test and urinalysis in patients participated in the clinical trial. MCH – mean corpuscular hemoglobin, HGB – hemoglobin, GLU – glucose, HCT – hematocrit, RBC – red blood cells, EOS – eosinophils, BIL-T – total bilirubin; hpf – high-power field.

*Normal values are presented according to the laboratory performed the analysis.
